# The Effect of Thyroid Dysfunction on the Cardiovascular Risk of Type 2 Diabetes Mellitus Patients in Ghana

**DOI:** 10.1155/2018/4783093

**Published:** 2018-02-01

**Authors:** Osei Sarfo-Kantanka, Fred Stephen Sarfo, Eunice Oparebea Ansah, Ishmael Kyei

**Affiliations:** ^1^Komfo Anokye Teaching Hospital, Kumasi, Ghana; ^2^Kwame Nkrumah University of Science and Technology, Kumasi, Ghana

## Abstract

**Background:**

Thyroid dysfunction is known to exaggerate the coronary heart disease (CHD) risk associated with type 2 diabetes mellitus (T2DM) among whites. The effect is yet to be studied among African populations.

**Methods:**

This is a cross-sectional study involving 780 T2DM patients enrolled in a diabetes clinic in Kumasi, Ghana. CHD risk was estimated using the Framingham and UKPDS risk scores. Risks were categorised as low (<10%), intermediate (10–19%), and high (≥20%). Associations between metabolic risk factors, thyroid dysfunction, and CHD risk were measured using Spearman's partial correlation analysis while controlling for age and gender. Differences were considered statistically significant at *p* < 0.05.

**Results:**

780 T2DM patients (57.7% females), mean ± SD age of 57.4 ± 9.4 was analysed. The median (IQR) 10-year CHD score estimated using the Framingham and UKPDS risk engines for males and females was 12 (8–20), 9.4 (5.7–13.4), *p* < 0.0001 and 3 (1–6), 5.8 (3.4–9.6), *p* < 0.0001, respectively. Positive correlation was found between CHD risk and HbA1c, total cholesterol, low-density lipoprotein cholesterol, systolic blood pressure, and thyroid stimulating hormone.

**Conclusion:**

The presence of thyroid dysfunction significantly increased the CHD risk associated with T2DM patients in Ghana.

## 1. Introduction

There has been a remarkable surge in the worldwide prevalence of type 2 diabetes mellitus (T2DM) over the past two decades. Per estimates by the International Diabetes Federation (IDF), at least 415 million people worldwide had diabetes mellitus in 2015 [1]. Countries in sub-Saharan Africa (SSA) will suffer significantly, with a projection of over 30 million cases expected to be recorded over the next three decades [2]. Ghana, like in most SSA countries, has been affected by profound escalations in the rates of all traditional vascular risk factors including diabetes, hypertension, dyslipidemia, and obesity [3]. Currently, an estimated 6% of Ghanaian urban population has been affected by diabetes [4].

Patients with diabetes compared to nondiabetic individuals bear up to six-fold higher risk of future coronary heart disease (CHD), equivalent to nondiabetic patients with preexisting cardiovascular disease [5–8]. Part of this excess risk found in diabetes patients is attributed to a higher prevalence of other cardiovascular disease (CVD) risk factors such as obesity, dyslipidemia, and hypertension.

Thyroid conditions represent the second commonest endocrine disorder seen in adults [9]. The coexistence of both T2DM and thyroid diseases can further increase the CHD risk associated with T2DM [10]. Like T2DM, thyroid disorders can have deleterious effects on the cardiovascular system; thyrotoxicosis leads to a hyperdynamic state with increased heart rate, left ventricular contractility, and systolic hypertension and may also be complicated by atrial fibrillation [11, 12]. Hypothyroidism, on the other hand, can enhance CHD risk through multiple interactions with indices like dyslipidemia and hypertension [13–16]. Identifying the CHD risk in T2DM patients with thyroid dysfunction will be beneficial in the end since evidence points to patients benefiting from aggressive strategies in CVD risk reduction [17].

Despite a well-known association of thyroid dysfunction with T2DM, no known studies have been conducted in sub-Saharan Africa to investigate the effect of such relationship on CHD risk of T2DM patients. Our objective in this study was to identify the effect of thyroid dysfunction on the CHD risk of Ghanaian T2DM patients attending an outpatient diabetes clinic using the well-validated Framingham risk [18] and the United Kingdom Prospective Diabetes Study Scores (UKPDS) [19].

## 2. Methods

### 2.1. Study Design and Setting

This study is a hospital-based cross-sectional study conducted at the Diabetes Clinic of the Komfo Anokye Teaching Hospital, a tertiary referral medical center in Kumasi, Ghana. Kumasi is the second largest city in Ghana, with an estimated population of 4 million inhabitants. The Diabetes Clinic was established in 1992 and runs throughout the working week receiving referrals for adults > 16 years with diabetes disorders from 6 out of the 10 administrative regions of Ghana with an estimated population of 10 million.

### 2.2. Study Participants

Consecutive T2DM patients attending the Diabetes Clinic at KATH were approached for enrollment into the study after obtaining informed consent. Patients were diagnosed with T2DM when they fulfilled the World Health Organization (WHO) diagnostic criteria for diabetes: an elevated fasting plasma glucose level (≥7 mmol/L) on two occasions, or oral glucose tolerance test ≥ 11.1 mmol/L [20], and age 30 years or older at the time of diagnosis, had not undergone insulin therapy for a year after diagnosis, and had no history of diabetic ketoacidosis. Using a structured validated questionnaire and a review of medical records, we obtained the sociodemographic and clinical information of all participants. Because of their confounding effects on thyroid function, we excluded pregnant women, patients on amiodarone, lithium, and long-term corticosteroids, and those with an acute illness and history of hospitalisation less than six months from the day of recruitment.

### 2.3. Consent and Ethical Approval

We obtained ethical approval from the Committee on Human Research Publication and Ethics (CHRPE) of the School of Medical Sciences, Kwame Nkrumah University of Science and Technology, and the Komfo Anokye Teaching Hospital (KATH), Kumasi, Ghana. All participants gave an informed consent with those unable to understand or sign the consent forms excluded.

### 2.4. Study Measurements

Patients were interviewed using a well-validated questionnaire to obtain information such as age, gender, smoking, and alcohol consumption habits. Current smoking status and alcohol intake status were ascertained from either the patient or a responsible relative. Smokers were identified by self-report as those who had smoked at least 10 sticks of cigarettes per day for six months or more or those who smoked daily for one year or more regardless of the number of cigarettes smoked per day [21]. A high alcohol intake was defined as ≥14 U per week for women and ≥21 U per week for men [22].

### 2.5. Physical Measurements

The weight of study subjects was measured in kilograms using a scale, and the height in centimeters using a Stadiometer with the patient standing at the anatomical position. The weight and height measurements were used to calculate the BMI. Subjects with BMI ≥ 30 kg/m^2^ were classified as obese [23].

Duplicate waist circumference (WC) measurements were taken, and the average was recorded using a plastic stretch-resistant anthropometric tape calibrated in centimeters and inches (made in Shanghai, China). The measurements were taken from the approximate midpoint between the lower margin of the last palpable rib and the superior border of the iliac crest at the level of the umbilicus in the midaxillary line with the participant erect and abdomen relaxed, arms by the side and heels together, and breathing normally. WC measurements > 80 cm and 94 cm were recorded as central obesity for females and males, respectively [23].

Blood pressure was measured thrice on the upper left arm using a validated automatic sphygmomanometer after at least 5 minutes of rest and the second and third readings averaged for analysis. Hypertension was diagnosed if the patient was on antihypertensive medications or if the patient had a systolic and diastolic blood pressure of 140/90 mmHg [24].

### 2.6. Laboratory Measurements of Biochemical Variables

Approximately ten milliliters (10 mL) of fasting venous samples was collected from each participant into Vacutainer tubes (Becton Dickinson, Rutherford, NJ) and Sequestrene bottles. Samples were manually processed and cryopreserved before transporting to the laboratory for analysis. Fasting plasma glucose (FPG), free thyroxine (FT4), free triiodothyronine (FT3), thyroid stimulating hormone (TSH), total cholesterol (TC), low-density lipoprotein cholesterol (LDL-C), high-density lipoprotein cholesterol (HDL-C), triglycerides (TG), urea, and creatinine levels were analysed on the serum obtained from each participant using a Cobras e 411 autoanalyzer® per manufacturer's instructions and previously described by Sarfo-Kantanka et al. [25].

Dyslipidemia was defined as a high TC, >5 mmol/L or LDL-cholesterol > 4 mmol/L, triglycerides > 1.7 mmol/L, or HDL-cholesterol < 1.30 mmol/L for women and <1.04 mmol/L for men or previous use of statin for dyslipidemia [26].

The reference ranges, intra-assay, and interassay coefficients of variation for thyroid hormones were as follows: TSH: 0.25–5.0 IU/mL, <2.1% and <2.4%; FT3: 3.7–10.4 pmol/L, 5.8% and 6.9% for FT3; and FT4: 7.5–21.1 pmol/L, 2.8% and 2.4%.

Thyroid function was classified as euthyroidism when free thyroxine (FT4), free triiodothyronine (FT3), and thyroid stimulating hormone (TSH) were within the normal range; clinical hypothyroidism when TSH level was greater than the upper limit of the reference range and FT4 or FT3 was lower than the lower limit of their reference ranges; subclinical hypothyroidism when TSH is greater than the upper limit of the reference range and FT4 and FT3 are within the normal range; clinical hyperthyroidism when TSH level was lower than the lower limit of the reference range and FT4 or FT3 was greater than the upper limit of their reference ranges; and subclinical hyperthyroidism when TSH level was lower than the lower limit of the reference range and FT3 and FT4 are within the normal range.

Glycated hemoglobin (HbA1c) measurements were performed using standardized high-performance liquid chromatography assay using Bio-Rad Variant. We followed the methodology described by Darko et al. [27].

### 2.7. Cardiovascular Risk Estimation

The Framingham risk engine and UKPDS questionnaire were administered to all eligible subjects and used to calculate the 10-year CHD risk of each participant. The Framingham risk questionnaire is a gender- and LDL-C-specific questionnaire and incorporates risk factors including age, sex, TC, HDL-C, SBP, DBP, smoking status, and presence of diabetes. UKPDS is an offline diabetes-specific risk engine used to estimate the 10-year CHD risk of an individual. The model is diabetes-specific and incorporates HbA1c, SBP, and lipid levels as risk factors, in addition to age, sex, ethnic group, smoking status, and time since diagnosis of diabetes. For the UKPDS risk estimation, participants were treated as African Caribbean. Estimated CHD risks were then categorised as low (<10%), intermediate (10–20%), and high (>20%).

### 2.8. Statistics

Data was entered into Microsoft Excel, and statistical analysis was performed using GraphPad Prism 5 software. Data normality was tested using D'Agostino and Pearson omnibus normality test. The statistical difference between means and medians was estimated by the Student *t*-tests and Mann‐Whitney *U* test. Associations were measured by coefficients of Spearman's partial correlation whiles controlling for age and gender. Differences were considered statistically significant at *p* < 0.05.

## 3. Results

### 3.1. Demographic and Clinical Characteristics

958 participants were approached, but 178 declined participation in the study. As shown in Table 1, 780 T2DM subjects, mean age 57.4 ± 9.4, were included in the analyses. Of these, 450 (57.7%) subjects were female. The median (IQR) age at diagnosis of participants was 50 (41–54), and the mean duration of T2DM was 9.8 ± 5.6 years. The predominant vascular risk factors identified included hypertension (77.9%), central obesity (74.6%), dyslipidemia (59.2%), and abnormal glycemic control, HbA1c > 7% (66.2%). Females had a significantly higher prevalence of central obesity, high BMI, and DBP. The median (IQR) 10-year CHD score estimated using the Framingham and UKPDS risk engines for males and females was 12 (8–20), 9.4 (5.7–13.4), *p* < 0.0001 and 3 (1–6), 5.8 (3.4–9.6), *p* < 0.0001, respectively. The median (IQR) CHD risk score estimated using the UKPDS engine was 4 (0.4–7): 9.4 (5.7–13.4) for males and 5.8 (3.4–9.6), *p* < 0.0001, for females.

### 3.2. CHD Score Based on Thyroid Status

**Figure 1:**
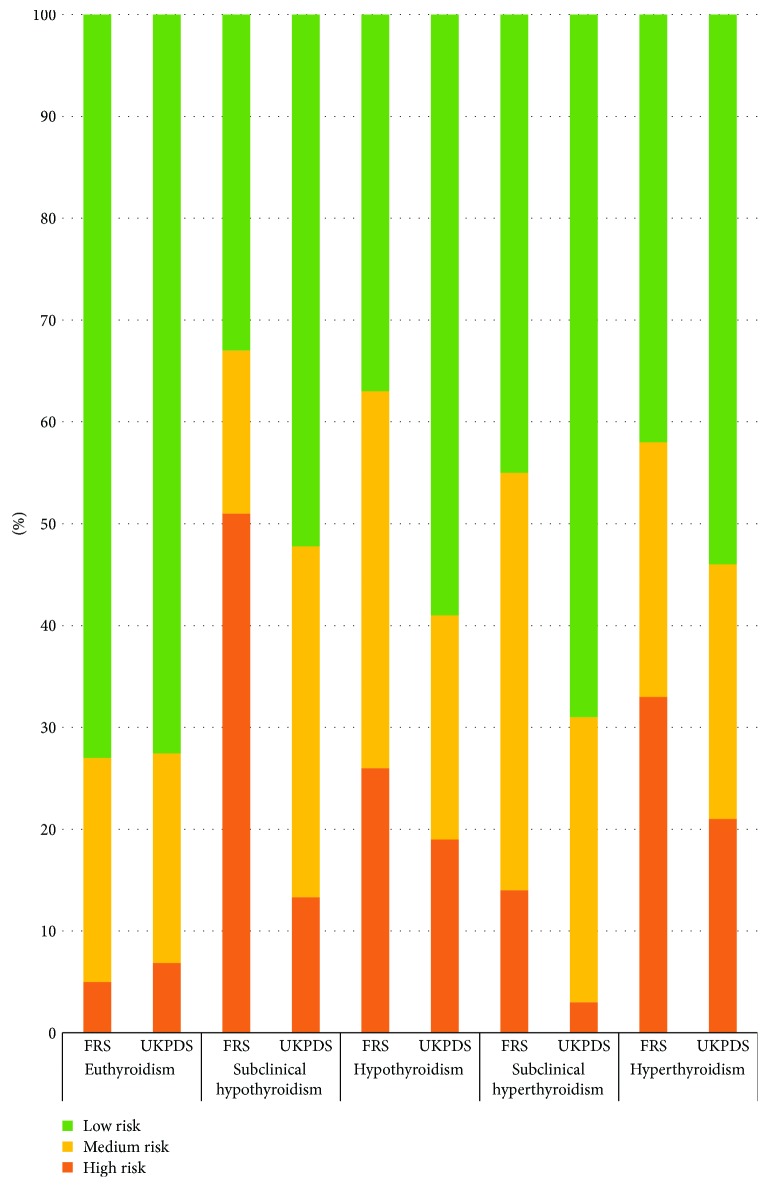
Variation of coronary heart disease risk per Framingham and UKPDS Scores.

Table 2 shows the median (IQR) Framingham risk score of T2DM patients with thyroid dysfunction significantly higher than for euthyroid T2DM patients: 11 (6–22) versus 4 (1–10), *p* < 0.0001. 8% of euthyroid T2DM subjects were identified as having high CHD risk using the Framingham risk score, 24% had intermediate risk, and 68% had low risk. Among those with thyroid dysfunction, 43% were identified as high risk, 28% as medium risk, and 25% as low risk. Of those identified with subclinical hyperthyroidism, 32% had high risk of CHD, 23% had an intermediate risk, and 44% had low risk. Of those with hyperthyroidism, 36% had high risk, 42% intermediate risk, and 22% low risk of CHD (Figure 1). In patients with subclinical hypothyroidism, 48% were classified as high risk, 29% as intermediate risk, and 23% as low risk; with hypothyroidism, 55% had high risk, 31% intermediate risk, and 14% low risk. The difference between the groups was highly significant (*p* < 0.0001). Using the UKPDS score, 4% of euthyroid T2DM subjects were identified as high risk, 44% as intermediate risk, and 52% as low risk. Among those with thyroid dysfunction, 26% were classified as high risk of CHD, 58% as intermediate risk, and 16% as low risk.

### 3.3. Correlates of Increased Cardiovascular Risk

As shown in Table 2, the demographic characteristics, vascular risk factor profile, and clinical features of T2DM subjects classified as having high CHD risk using the Framingham risk score were older, had female gender predisposition, older age at diagnosis, obese (general and central), had poorer glycemic control and lipid profiles, higher blood pressures, and higher rates of alcohol abuse compared to those with intermediate or low CHD risks.

#### 3.3.1. Association between Biochemical and Physical Characteristics and Coronary Heart Disease Risk in T2DM Patients

A strong positive correlation was found between high Framingham CHD risk and HbA1c (*r* = 0.51, *p* < 0.04), TC (*r* = 0.49, *p* < 0.0001), LDL-C (*r* = 0.37, *p* < 0.0001), and TSH (*r* = 0.27, *p* = 0.01). Under the UKPDS scoring, a strong association was found between CHD risk and HbA1c (*r* = 0.42, *p* < 0.0001) and TSH (*r* = 0.32, *p* < 0.02) (Table 3).

## 4. Discussion

We have found in this study that T2DM subjects in Ghana possess high prevalence levels of modifiable vascular risk factors: 74.5% had central obesity, 77.9% had hypertension, and 59.2% had dyslipidemia. In addition, 30.0% abused alcohol and 7.4% smoked cigarette. In general terms, glycemic control was poorer among male T2DM subjects showing poorer controls compared to females. Females had higher prevalence of some important vascular risk factors. This is explained by the fact that female T2DM patients especially those who are menopausal have worse CVD risk factor profiles and mortalities compared to their male counterparts [28, 29]. This was illustrated by a meta-analysis of 37 prospective cohort studies where the risk of fatal CHD was 50% higher in female T2DM patients compared to males [30]. The reason for this postmenopausal T2DM female predilection towards increased CHD risk is multifactorial. Principally, it has been demonstrated that chronic hyperglycemia alters the estrogen-related protective mechanisms that leave premenopausal woman at a lower CVD risk compared to males. This leads to notable adverse changes in CVD risk factors leading to enhanced atherogenesis in females even premenopausal [31, 32]. Additionally, postmenopausal T2DM females have been shown to possess a relatively heavier risk factor burden [32] as well as a significant array and involvement of inflammatory factors [33], smaller size of coronary vessels, and disordered angiogenesis [34, 35]. The removal of the protective effect of female sex on the risk of CHD in people with diabetes warrants aggressive screening and treatment of females with this risk [36].

The proportion of patients with high CHD risk (18.7%) recorded in this present study is comparable to that found in similar studies involving African American [37] and non-Hispanic white T2DM subjects [38] who were assessed using the Framingham risk scoring. However, among Brazilian T2DM subjects where UKPDS was used in risk assessment, a significantly higher CHD risk was recorded [39]. It is unknown whether these differences observed in CHD risk prevalence are due to the different methods used for assessment and study sample sizes. A high risk of CHD was seen among Caucasians and South Americans [38, 39]. This applies particularly to men, where both morbidity and mortality from heart disease are about half that of the general population [40]. Data on African Americans from the United States conflict this finding, showing higher CHD rates than US whites [41]. This appears to be in part explained by the degree of acculturation and miscegenation, as birthplace within or outside the USA was a strong determinant of mortality risk [35]. Furthermore, African men have been shown to be less centrally obese than their Caucasian counterparts [37].

Thus, with this present study, which to the best of our knowledge is the first among West Africans, we have shown a higher CHD risk in T2DM subjects with thyroid dysfunction compared with those who were euthyroid. This we attribute to the significantly higher risk factor burden of CHD seen in T2DM subjects with thyroid dysfunction compared to their euthyroid counterparts recorded in the study. Moreover, this effect is better explained by the higher CHD risk seen among patients with hypothyroidism compared to those with other forms of thyroid dysfunction and euthyroidism. It is well known that patients with hypothyroidism possess significant incidence of inflammatory factors, hypercholesterolemia, and diastolic blood pressures compared to those with other forms of thyroid dysfunction [38].

A significantly positive linear association found in T2DM subjects between TSH and CHD risk could be attributed to the increasing association of thyroid dysfunction with high inflammatory markers, dyslipidemia, and poor glycemic control [39]. It has been postulated that high inflammatory state induced by thyroid dysfunction produces oxidative stress, insulin resistance, cell apoptosis, vascular endothelial injury, platelet activation, and neuroendocrine disorders which all promote the development of atherosclerosis [40, 41]. For this reason, it is now recognised that subclinical hypothyroidism together with hypothyroidism are risk factors for increased CHD risk and mortality in the general population as well as diabetics even after accounting for traditional vascular risk factors [42].

Some inherent limitations were associated with this study including our sample size composition. We had fewer males with T2DM relative to females which may have influenced the results. Moreover, confounders such as physical activity and dietary patterns were not explored in this study even though they could have influenced the CHD risk. Secondly, the cross-sectional study design means that the associations observed between thyroid dysfunction and CHD risk could not be interpreted as causal relationships.

## 5. Conclusion

We conclude that our T2DM patients with thyroid dysfunction compared to their euthyroid counterparts have a significantly higher CHD risk. A significant association of CHD risk has been shown with HbA1c and dyslipidemia. Physicians taking care of these patients should have a high index of suspicion for thyroid dysfunction in patients with high CVD risk.

## Figures and Tables

**Table 1:** Demographic and clinical characteristics of 780 T2DM participants enrolled for the study.

Test parameters	Total	Male	Female	*p* value
Number (%)	780 (100)	330 (42.3)	450 (57.7)	
Age, mean ± SD	57.4 ± 9.4	57.8 ± 9.5	57.3 ± 9.3	0.65
Age at diagnosis, median (IQR)	50 (43–56)	51 (43–60)	50 (43–78)	0.08
Duration of diabetes, mean ± SD	9.8 ± 5.6	9.9 ± 3.2	9.6 ± 8.9	0.09
Body mass index (kg/m), median (IQR)	27.5 (24.8–30.9)	27.0 (24–30.3)	27.9 (25.1–31.5)	0.04^∗^
General obesity (%)	262 (33.6)	92 (27.9)	170 (37.8)	0.04^∗^
Waist circumference, cm, median (IQR)	97 (87–105)	94 (84–101)	98 (90–106)	<0.001^∗^
Central obesity, *n* (%)	582 (74.6)	156 (47.3)	426 (94.7)	<0.001^∗^
Fasting blood glucose, median (IQR)	8.8 (6.7–12.6)	8.6 (6 .7–12.2)	9.1 (6.8–13.5)	0.4
Hba1c (%), median (IQR)	8.1 (6.8–9.6)	8.1 (6.6–9.8)	8.1 (6.9–9.6)	0.92
Abnormal glycemic control (>7%)	716 (66.2)	220 (66.7)	286 (63.6)	0.85
Total cholesterol mmol/L, median (IQR)	5.0 (4.1–6.0)	5 (4.2–5.9)	5 (4–6.1)	0.63
Hypercholesterolemia, *n* (%)	400 (51.3)	164 (49.7)	236 (52.4)	0.15
Triglycerides (mmol/L), median (IQR)	1.2 (0.9–1.6)	1.16 (0.85–1.6)	1.24 (0.94–1.57)	0.22
Hypertriglyceridemia, *n* (%)	170 (21.8)	66 (20.0)	104 (23.1)	0.44
LDL-C, median (IQR)	3.2 (2.1–3.9)	3.1 (2.2–3.8)	3.2 (2 .1–4.0)	0.92
High LDL, *n* (%)	470 (60.3)	186 (56.4)	284 (63.1)	0.18
HDL-C, median (IQR)	1.2 (1–1.5)	1.1 (1–1.4)	1.2 (1.0–1.5)	0.2
Low HDL-C, *n* (%)	54 (6.9)	32 (9.6)	22 (4.9)	0.007^∗^
Dyslipidemia, *n* (%)	462 (59.2)	196 (59.4)	266 (59.1)	0.12
Systolic blood pressure, median (IQR)	140 (130–150)	140 (129–150)	140 (130–150)	0.43
Diastolic blood pressure, median (IQR)	80 (70–90)	80 (70–90)	80 (80–90)	0.01^∗^
Hypertension, *n* (%)	602 (77.9)	272 (82.4)	330 (73.3)	0.03^∗^
Alcohol, *n* (%)	234 (30.0)	200 (60.6)	34 (7.6)	<0.001^∗^
Current smokers, *n* (%)	58 (7.4)	56 (17.0)	2 (0.4)	<0.001^∗^
Framingham CHD risk, %, median (IQR)	6 (2–12)	12 (8–20)	3 (1–6)	<0.001^∗^
UKPDS CHD risk score, median (IQR)	4 (0.4–7)	9.4(5.7–13.4)	5.8 (3.4–9.6)	<0.001^∗^
Subclinical hypothyroidism	64 (8.2)	14 (4.2)	50 (11.1)	0.02^∗^
Subclinical hyperthyroidism	22 (2.8)	14 (4.2)	8 (1.8)	<0.001
Hypothyroidism	12 (1.6)	2 (0.6)	10 (2.2)	0.08
Hyperthyroidism	4 (0.5)	2 (0.6)	2 (0.4)	0.21
Euthyroidism	678 (86.9)	298 (90.3)	380 (84.4)	0.02

CHD: coronary heart disease; HbA1c: glycated hemoglobin; HDL-C: high-density lipoprotein cholesterol; IQR: interquartile range; LDL-C: low-density lipoprotein cholesterol; UKPDS: United Kingdom Prospective Diabetes Study; SD: standard deviation. ^∗^*p* < 0.05 statistically significant.

**Table 2:** Demographic and clinical characteristics of T2DM participants based on CHD risk.

Test parameters	Total	CHD risk			*p*
		Low	Moderate	High	
Number (%)	780 (100)	424 (54.4)	210 (26.9)	146 (18.7)	
Age, mean ± SD	59.2 ± 8.6	56.6 ± 7.9	61.6 ± 8.5	62.9 ± 8.4	<0.0001^∗^
Female gender, *n* (%)	450 (57.7)	326 (76.9)	124 (59.0)	52 (35.6)	<0.0001^∗^
Age at diagnosis, mean ± SD	51.3 ± 9.0	47.4 ± 7.2	52.8 ± 7.9	54.2 ± 10.2	<0.0004^∗^
BMI, median (IQR)	27 (24.1–30.8)	27 (24–29.5)	26.7 (23–30.9)	27 (24–30.9)	0.37
Waist circumference, median (IQR)	98 (88–108)	87.3 (95.5–105)	107 (99–114)	95 (85–109)	<0.0001^∗^
Central obesity, *n* (%)	582 (74.6)	256 (60.4)	190 (90.5)	136 (93.2)	<0.0001^∗^
Fasting glucose, median (IQR)	8.6 (6.7–13.0)	8.2 (6.7–12.9)	8.6 (6–12.5)	9.4 (7.1–14)	0.37
Hba1c (%), median (IQR)	8.2 (7–9.8)	7.9 (6.9–9.5)	8.7 (6.9–9.9)	8.4 (7.3–10)	<0.0001^∗^
Abnormal glycemic control (>7%)	516 (66.2)	232 (54.7)	160 (76.2)	124 (84.9)	<0.001^∗^
Total cholesterol, median (IQR)	5.3 (4.3–6.65)	5.0 (4.1–5.9)	5.7 (4.7–6.9)	6.1 (6–7.9)	<0.0001^∗^
Hypercholesterolemia, *n* (%)	400 (51.3)	196 (46.2)	112 (53.3)	92 (63.0)	<0.0001^∗^
Triglycerides, median (IQR)	0.95 (1.22–1.6)	1.2 (0.9–1.5)	1.2 (1.0–1.7)	1.4 (1.0–1.7)	0.06
LDL-C, median (IQR)	3.3 (2.3–4.2)	3.2 (2.2–3.8)	3.6 (2.6–4.4)	4.19 (3.2–5)	<0.0001^∗^
High LDL, *n* (%)	470 (60.3)	216 (50.9)	136 (64.8)	118 (80.8)	<0.0001^∗^
HDL-C, median (IQR)	1.2 (1–1.43)	1.2 (1–1.5)	1.2 (1–1.4)	1.1 (1–1.32)	0.14
Low HDL-C, *n* (%)	108 (13.8)	26 (6.1)	38 (18.1)	44 (30.1)	<0.0001^∗^
Dyslipidemia, *n* (%)	462 (59.2)	212 (50.0)	124 (59.1)	126 (86.3)	<0.00001^∗^
Systolic blood pressure, median (IQR)	140 (130–152)	140 (130–150)	140 (130–154)	148 (130–160)	0.14
Diastolic blood pressure, median (IQR)	80 (80–90)	80 (80–90)	80 (70–90)	80 (80–90)	0.78
Hypertension, *n* (%)	602 (77.9)	304 (71.7)	158 (75.2)	140 (95.9)	<0.001^∗^
Alcohol, *n* (%)	234 (30.0)	108 (25.5)	62 (29.5)	64 (43.8)	<0.0001^∗^
Current smokers, *n* (%)	58 (7.4)	26 (6.1)	16 (7.6)	18 (12.3)	<0.0001^∗^

CHD: coronary heart disease; HbA1c: glycated hemoglobin; HDL-C: high-density lipoprotein cholesterol; IQR: interquartile range, LDL-C: low-density lipoprotein cholesterol; SD: standard deviation. ^∗^*p* < 0.05 statistically significant.

**Table 3:** Pearson bivariate correlation between ten-year coronary heart disease risk and independent variables.

Test parameters	Framingham	UKPDS
Waist circumference	0.12 (0.061)	−0.08 (0.42)
Glycated hemoglobin, HbA1c	0.51 (0.041)	0.42 (<0.0001)
Total cholesterol	0.49 (0.0001)	0.14 (<0.51)
Low-density lipoprotein cholesterol	0.37 (<0.0001)	0.10 (0.04)
High-density lipoprotein cholesterol	0.02 (0.21)	−0.06 (0.12)
Triglycerides	0.12 (0.02)	0.11 (0.06)
Systolic blood pressure	0.12 (0.12)	0.10 (0.04)
Thyroid stimulating hormone	0.27 (0.0006)	0.32 (0.02)
Free triiodothyronine	0.07 (0.23)	0.10 (0.34)
Free thyroxine	0.11 (0.09)	0.13 (0.15)

## Abbreviations

CHD: Coronary heart disease
CVD: Cardiovascular disease
FT3: Free triiodothyronine
FT4: Free thyroxine
HbA1c: Glycated hemoglobin
HDL: High-density lipoprotein cholesterol
LDL-C: Low-density lipoprotein cholesterol
TC: Total cholesterol
TG: Triglycerides
T2DM: Type 2 diabetes mellitus
UKPDS: United Kingdom Prospective Diabetes Study.
